# Subclinical thyroid dysfunction and chronic kidney disease: a nationwide population-based study

**DOI:** 10.1186/s12882-023-03111-7

**Published:** 2023-03-22

**Authors:** Hye Jeong Kim, Sang Joon Park, Hyeong Kyu Park, Dong Won Byun, Kyoil Suh, Myung Hi Yoo

**Affiliations:** 1 Division of Endocrinology and Metabolism, Department of Internal Medicine, Soonchunhyang University Hospital, Soonchunhyang University College of Medicine, 59 Daesagwan-ro, Yongsan-gu, Seoul, 04401 Republic of Korea; 2 Elim Thyroid Clinic, Seoul, Korea

**Keywords:** Thyroid function, Hypothyroidism, Chronic kidney disease, KNHANES

## Abstract

**Background:**

Chronic kidney disease (CKD) has a significant impact on global health. Studies have shown that subclinical thyroid dysfunction may be related to CKD, but the association between subclinical thyroid dysfunction and CKD in the general population is unclear. We aimed to evaluate the risk of CKD according to thyroid function status in a large cohort.

**Methods:**

We analyzed data from a nationwide, population-based, cross-sectional survey (KNHANES VI). A total of 3,257 participants aged ≥ 19 years who underwent thyroid and kidney function assessments were included in this study. CKD was defined as an estimated glomerular filtration rate < 60 mL/min/1.73 m^2^ and/or urine albumin-creatinine ratio ≥ 30 mg/g. The risk of CKD according to thyroid function status was assessed using logistic regression, adjusted for potential confounders.

**Results:**

Overall, 6.7% of the participants had CKD. There were no significant differences in thyroid-stimulating hormone and free thyroxine levels between the groups with and without CKD. The proportion of participants with CKD was significantly different among the thyroid function status groups (p = 0.012) and tended to increase significantly in the following order: subclinical hyperthyroidism (1.5%), euthyroidism (6.6%), and subclinical hypothyroidism (12.6%) (p for trend < 0.001). Subclinical hypothyroidism was a significant risk factor for CKD, even after adjusting for sex, age, household income, education, smoking, alcohol consumption, walking activity, abdominal obesity, hypertension, low high-density lipoprotein cholesterol, elevated triglycerides, hyperglycemia, free thyroxine, and thyroid-peroxidase anibody (odds ratio 2.161, 95% confidence interval 1.032–4.527, p = 0.041).

**Conclusion:**

Subclinical hypothyroidism is an independent predictor of CKD in the general population.

**Supplementary Information:**

The online version contains supplementary material available at 10.1186/s12882-023-03111-7.

## Introduction

Chronic kidney disease (CKD) is a complex and multifaceted disease that causes renal dysfunction and progression to end-stage kidney disease. CKD has a significant impact on global health as a direct cause of morbidity and mortality and is an important risk factor for cardiovascular disease (CVD) [1]. The prevalence of CKD is high and has increased in all age groups over the past few decades due to an increased aging population and prevalence of diabetes, obesity and hypertension [2–5]. This trend is particularly concerning given the high clinical and economic burden associated with the progression of CKD [1]. Thus, early detection and management of risk factors for CKD is very important.

The kidney plays a role in the regulation of metabolism and elimination of thyroid hormones [6]. Thyroid hormones are also necessary for the growth and development of the kidney and for the maintenance of water and electrolyte homeostasis [6]. Both hypothyroidism and hyperthyroidism affect renal blood flow, glomerular filtration, tubular function, electrolytes homeostasis, electrolyte pump functions, and kidney structure [6] and lead to increased risk of CVD [7]. Although some studies found no association between subclinical thyroid dysfunction and CKD [8–11], other studies have identified subclinical thyroid dysfunction as a risk factor for CKD [12–15]. Data on subclinical thyroid dysfunction and the risk of CKD in the general population are inconclusive, and more research is needed to fully understand the potential impact of subclinical thyroid dysfunction on kidney health.

In the present study we aimed to clarity the risk of CKD according to thyroid function status in a large cohort.

## Methods

### Study population

This study used data from the Korea National Health and Nutrition Examination Survey (KNHANES) VI (2013–2014). The KNHANES is a nationwide, cross-sectional survey conducted by the Korean Centers for Disease Control and Prevention (KCDC) to assess the health and nutritional status of the Korean population [16]. The study participants were selected using stratified multistage cluster sampling and housing census data. Among the participants, approximately 2400 individuals (1/3 of the participants aged ≥ 10 years) were selected for laboratory tests of serum thyroid-stimulating hormone (TSH) and free thyroxine (fT4) using stratified subsampling according to sex and age in each year [16].

**Fig. 1:**
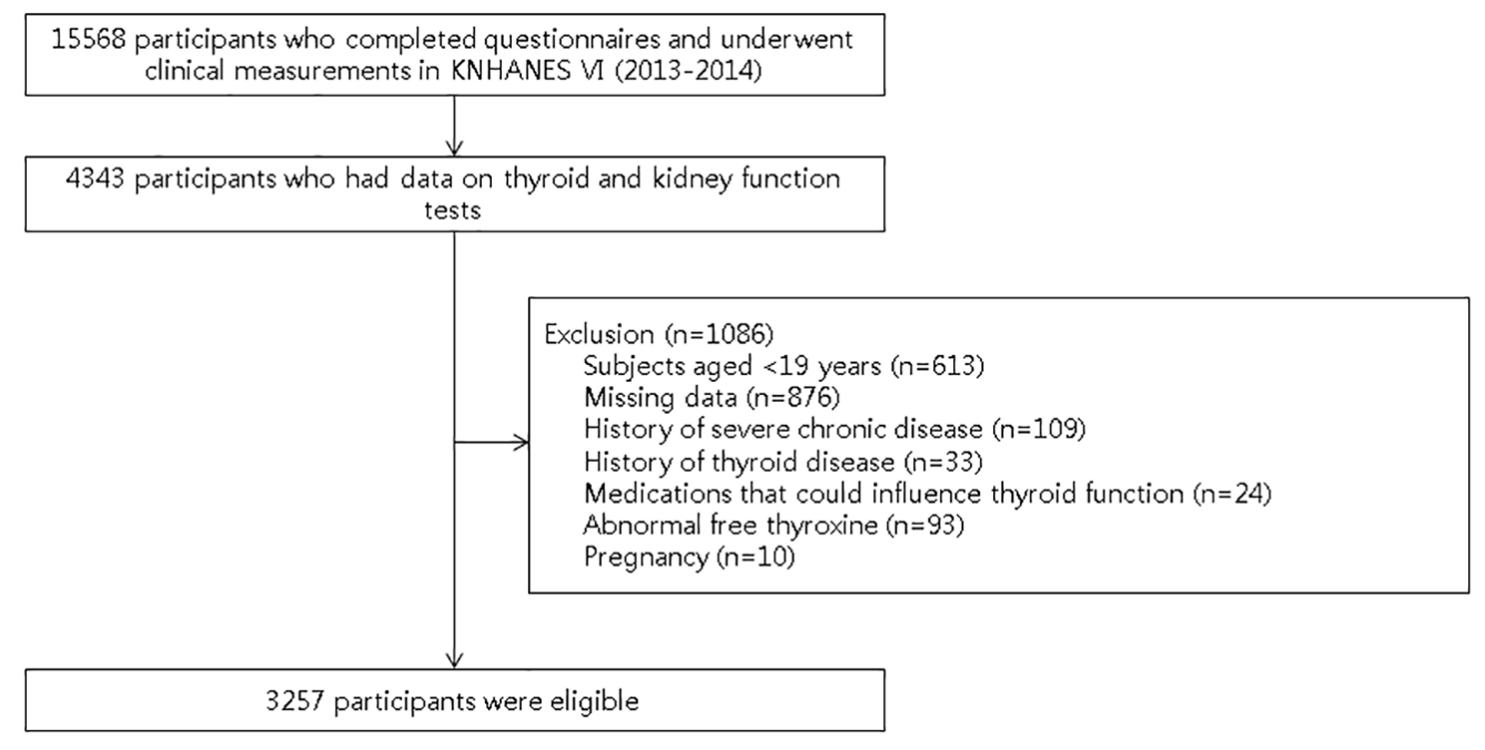
Flow chart of the study population. KNHANES, Korean National Health and Nutrition Examination Survey

There were 15,568 study participants, of which 4,343 underwent both thyroid [TSH, fT4 and thyroid-peroxidase antibody (TPOAb)] and kidney (serum creatinine, urine albumin, and urine creatinine) function tests. Participants were excluded for the following reasons: (1) age < 19 years (n = 613); (2) missing data (questionnaires about household income, education, smoking, alcohol, or exercise; and history of diabetes, hypertension, thyroid disease, cancer, or liver cirrhosis) (n = 876); (3) history of severe chronic disease, such as any type of cancer or liver cirrhosis (n = 109); (4) history of thyroid disease, including hyperthyroidism, hypothyroidism, benign thyroid nodules, or Hashimoto’s thyroiditis (n = 33); (5) use of medication that could influence thyroid function, including radioactive iodine therapy, antithyroid drugs, and/or thyroid hormones (n = 24); (6) abnormal fT4 levels (< 0.89 ng/dL or > 1.76 ng/dL) (n = 93); and (7) pregnancy (n = 10). Several participants met more than two of the exclusion criteria. Finally, 3,257 participants were included in the analysis (Fig. 1).

### Clinical and anthropometric measurements

Household income level, education level, smoking status, alcohol consumption, and walking activity were assessed using a self-reported questionnaire. Household income levels were categorized into four groups according to income quartiles: low, middle-low, middle-high, and high. Educational attainment was classified into four groups: elementary school or lower, middle school, high school, and college or higher. Regarding smoking status, participants were categorized as current, former, or never smokers [17]. For alcohol consumption, participants were classified as excessive (> 21 drinks/week in men and > 14 drinks/week in women) [18], moderate (≤ 21 drinks/week in men and ≤ 14 drinks/week in women), or never drinkers [19]. Walking activity was categorized as active or inactive. Participants were considered active if they walk least 5 days weekly for at least 10 min per day [20].

Physical examinations, including height, weight, waist circumference, and blood pressure, were performed as described in a previous study [21]. Body mass index (BMI) was calculated as the weight in kilograms divided by the height in meters squared (kg/m^2^). According to the World Health Organization standards for Asians, BMI was categorized into the following categories: underweight (< 18.5 kg/m^2^), normal weight (18.5–22.9 kg/m^2^), overweight (23-24.9 kg/m^2^) and obese (≥ 25 kg/m^2^) [22].

### Laboratory assay

Laboratory assays for triglycerides, high-density lipoprotein (HDL) cholesterol, fasting glucose, glycated hemoglobin (HbA1c), and thyroid function tests were performed as described in a previous study [20]. Serum and urine creatinine levels were measured using a Jaffe rate-blanked and compensated method with a Hitachi Automatic Analyzer 7600 − 210 (Hitachi Ltd, Tokyo, Japan). The estimated glomerular filtration rate (eGFR) calculated using the Chronic Kidney Disease Epidemiology Collaboration (CKD-EPI) equation [23]: for females with a serum creatinine level ≤ 0.7 mg/dL, eGFR = 144 × (Serum creatinine/0.7)^−0.329^ × (0.993)^Age^; for females with a serum creatinine level > 0.7 mg/dL, eGFR = 144 × (Serum creatinine/0.7)^−1.209^ × (0.993)^Age^; for males with a serum creatinine level ≤ 0.9 mg/dL, eGFR = 141 × (Serum creatinine/0.9)^−0.411^ × (0.993)^Age^; for males with a serum creatinine level > 0.9 mg/dL, eGFR = 141 × (Serum creatinine/0.9)^−1.209^ × (0.993)^Age^. Urine albumin was measured in random urine samples using a turbidimetric assay with a Hitachi Automatic Analyzer 7600 (Hitachi Ltd). The urine albumin-creatinine ratio (ACR) was reported as milligrams of albumin per gram of creatinine (mg/g).

### Definitions

Euthyroidism was defined as serum TSH (reference range, 0.62–6.68 mIU/L) [16] and fT4 (laboratory reference range, 0.89–1.76 ng/dL) levels within normal reference ranges. Subclinical hyperthyroidism was defined as TSH levels < 0.62 mIU/L and normal fT4 levels, and subclinical hypothyroidism was defined as TSH levels > 6.68 mIU/L and normal fT4 levels.

CKD was defined as an eGFR < 60 mL/min/1.73m^2^ and/or ACR ≥ 30 mg/g [24].

### Statistical analysis

Weighted sample values were used for analysis to reflect the stratified multistage probability sampling design of the KNHANES VI. Owing to a skewed distribution, a logarithmic transformation of TSH values was used in the analysis. Continuous variables are reported as means (standard error), and categorical variables are presented as weighted percentages (%). The demographic and biochemical characteristics of the study population with respect to CKD were compared using a general linear model for continuous variables and the chi-square test for categorical variables. The prevalence of metabolic syndrome components and CKD markers according to thyroid function status was compared using a general linear model or the chi-square test. Complex sample logistic regression analyses were used to determine the risk of CKD based on thyroid function status. The results are expressed as odds ratios (ORs) with 95% confidence intervals (CIs). All p values and 95% CI for OR were corrected using the Bonferroni method due to multiple testing. Additional adjustments were made for confounding variables, such as age, sex, household income, education, smoking, alcohol consumption, walking activity, abdominal obesity, hypertension, low HDL cholesterol, elevated triglycerides, hyperglycemia, fT4, and TPOAb.

All statistical analyses were performed using SPSS Statistics version 26.0 (IBM Corp., Chicago, IL, USA). All tests were two sided, and a p value of < 0.05 was considered statistically significant.

## Results

The baseline clinical and biochemical characteristics of the 3,257 participants are presented in Table 1. In the cohort, 54.1% of participants were men, with a mean age of 44.10 (0.28) years.

**Table 1:** Baseline characteristics of participants according to chronic kidney disease status

Variables	Chronic kidney disease			Overall (unweighted N = 3,257) (weighted N = 18,222,051)
	No (n = 3,033, 93.3%)	Yes (n = 224, 6.7%)	p value	
Sex			0.773	
Male (%)	54.0	55.1		54.1
Female (%)	46.0	44.9		45.9
Age (years)	43.34 (0.28)	54.69 (1.02)	< 0.001	44.10 (0.28)
Household income level			< 0.001	
Quartile 1 (lowest) (%)	11.6	20.2		12.2
Quartile 2 (%)	27.1	33.1		27.5
Quartile 3 (%)	29.7	30.3		29.8
Quartile 4 (highest) (%)	31.5	16.4		30.5
Education level			< 0.001	
Elementary school or lower (%)	13.2	30.0		14.3
Middle school (%)	9.4	15.4		9.8
High school (%)	40.9	34.0		40.4
College or higher (%)	36.5	20.6		35.4
Smoking			0.042	
Current (%)	27.1	22.8		26.8
Former (%)	18.6	26.2		19.1
Never (%)	54.3	51.0		54.1
Alcohol consumption			0.149	
Excessive (%)	10.4	10.2		10.4
Moderate (%)	82.0	78.0		81.7
Never (%)	7.6	11.9		7.9
Walking activity			0.497	
Active (%)	55.5	52.5		55.3
Inactive (%)	44.5	47.5		44.7
BMI^a^ (kg/m^2^)	23.74 (0.08)	24.92 (0.34)	0.001	23.82 (0.08)
Underweight (%)	4.0	2.9	0.003	3.9
Normal weight (%)	39.5	26.3		38.6
Overweight (%)	24.6	27.2		24.8
Obese (%)	31.9	43.6		32.6
Waist circumference				
Male (cm)	83.57 (0.25)	88.19 (1.07)	< 0.001	83.88 (0.24)
Female (cm)	76.88 (0.34)	80.44 (1.20)	0.003	77.11 (0.33)
Abdominal obesity^b^ (%)	28.6	41.6	< 0.001	29.5
Systolic BP (mmHg)	115.36 (0.34)	127.83 (1.63)	< 0.001	116.20 (0.35)
Diastolic BP (mmHg)	75.31 (0.23)	78.89 (0.89)	< 0.001	75.55 (0.23)
Hypertension^c^	29.8	65.1	< 0.001	32.2
Total cholesterol (mg/dL)	187.29 (0.79)	201.49 (2.96)	< 0.001	188.24 (0.79)
HDL cholesterol				
Male (mg/dL)	48.14 (0.30)	47.24 (1.15)	0.443	48.08 (0.29)
Female (mg/dL)	54.25 (0.35)	50.73 (1.18)	0.006	54.02 (0.33)
Low HDL cholesterol^d^ (%)	29.8	40.4	0.008	30.5
Triglycerides (mg/dL)	137.14 (2.49)	184.92 (11.69)	< 0.001	140.34 (2.49)
Elevated triglycerides^e^ (%)	29.4	50.2	< 0.004	30.8
Fasting glucose (mg/dL)	97.03 (0.42)	111.06 (2.47)	< 0.001	97.97 (0.44)
HbA1c (%)	5.73 (0.02)	6.41 (0.11)	< 0.001	5.78 (0.02)
Hyperglycemia^f^ (%)	28.2	56.2	< 0.001	30.1
eGFR (mL/min/1.73 m^2^)	98.85 (0.30)	84.80 (1.95)	< 0.001	97.90 (0.33)
ACR (mg/g)	5.37 (0.11)	153.07 (27.90)	< 0.001	15.26 (2.05)
TSH (mIU/L)	2.23 (0.04)	2.22 (0.19)	0.178	2.23 (0.04)
fT4 (ng/dL)	1.24 (0.01)	1.21 (0.01)	0.064	1.23 (0.01)
TPOAb (IU/mL)	26.90 (3.24)	30.91 (8.43)	0.655	27.17 (3.08)
Positivity of TPOAb^g^ (%)	6.1	8.5	0.236	6.2

BMI, body mass index; BP, blood pressure; HDL, high-density lipoprotein; HbA1c, glycated hemoglobin; eGFR, estimated glomerular filtration rate; ACR, albumin-creatinine ratio; TSH, thyroid-stimulating hormone; fT4, free thyroxine; TPOAb, anti-thyroid peroxidase antibody

^a^Underweight (< 18.5 kg/m^2^), normal weight (18.5–22.9 kg/m^2^), overweight (23-24.9 kg/m^2^) and obese (≥ 25 kg/m2); ^b^Waist circumference ≥ 90 cm in men and ≥ 80 cm in women; ^c^BP ≥ 130/85 mmHg or undergoing treatment with antihypertensive medication; ^d^HDL cholesterol < 40 mg/dL in men and < 50 mg/dL in women; ^e^Triglycerides ≥ 150 mg/d; ^f^Fasting glucose levels ≥ 100 mg/dL, HbA1c ≥ 6.5% or currently on antidiabetic medication; ^g^TPOAb ≥ 34.0 IU/mL.

Among the study participants, 6.7% were diagnosed with CKD. Participants with CKD were older, had relatively low household income and education levels, and were more likely to be former smokers than those without CKD. They also had higher BMI, waist circumference, BP, total cholesterol, triglycerides, fasting glucose, HbA1c, and ACR, but lower HDL cholesterol in women and eGFR. However, there were no significant differences between the groups in terms of sex, alcohol consumption, walking activity, and levels of HDL cholesterol in men, TSH, fT4, and TPOAb.

We further investigated the metabolic syndrome components and CKD markers according to thyroid function status (Table 2). For metabolic syndrome components, the proportion of participants with low HDL cholesterol levels was significantly different among the three groups (p = 0.003). There were no significant differences among the groups with respect to abdominal obesity, hypertension, elevated triglycerides and hyperglycemia.

**Table 2:** Comparison of the prevalence of metabolic syndrome components and chronic kidney disease markers according to thyroid function status

Variables	Thyroid function status			p value
	Subclinical hyperthyroidism (n = 77, 2.5%)	Euthyroidism (n = 3,069, 94.0%)	Subclinical hypothyroidism (n = 111, 3.5%)	
Abdominal obesity^a^ (%)	37.7	29.1	35.1	0.206
Hypertension^b^ (%)	29.2	32.2	34.6	0.815
Low HDL cholesterol^c^ (%)	47.0	29.7	40.0	0.003
Elevated triglycerides^d^ (%)	30.0	30.6	35.9	0.578
Hyperglycemia^e^ (%)	27.9	30.1	30.7	0.928
eGFR (mL/min/1.73 m^2^)	101.21 (2.13)	97.90 (0.35)	95.61 (1.49)	0.107
eGFR < 60 mL/min/1.73 m^2^ (%)	1.5	1.5	1.5	0.999
ACR (mg/g)	5.92 (0.68)	15.66 (2.17)	11.16 (1.80)	< 0.001
ACR ≥ 30 mg/g (%)	NA	5.8	11.1	0.020
CKD^f^ (%)	1.5	6.6	12.6	0.012

MetS, metabolic syndrome; HDL, high-density lipoprotein; BP, blood pressure; eGFR, estimated glomerular filtration rate; ACR, albumin-creatinine ratio; CKD, chronic kidney disease

^a^Waist circumference ≥ 90 cm in men and ≥ 80 cm in women; ^b^Blood pressure ≥ 130/85 mmHg or antihypertensive medication; ^c^HDL cholesterol < 40 mg/dL in men and < 50 mg/dL in women; ^d^Triglycerides ≥ 150 mg/dL; ^e^Fasting glucose ≥ 100 mg/dL, glycated hemoglobin (HbA1c) ≥ 6.5% or antidiabetic medication; ^f^eGFR < 60 mL/min/1.73 m^2^ or ACR ≥ 30 mg/g.

Regarding CKD markers, ACR levels (p < 0.001) and the proportion of participants with ACR ≥ 30 mg/g (p = 0.020) were significantly different among the three groups. The proportion of participants with CKD differed significantly among the three groups and tended to increase significantly in the following order: subclinical hyperthyroidism (1.5%), euthyroidism (6.6%) and subclinical hypothyroidism (12.6%) (p = 0.012, p for trend < 0.001). There were no significant differences among the three groups with respect to eGFR and the proportion of participants with eGFR < 60 mL/min/1.73 m^2^.

The results of the logistic regression analyses for the CKD risk based on thyroid function status are shown in Table 3. Participants with subclinical hypothyroidism had a significantly greater risk of CKD than those with euthyroidism (OR 2.039, 95% CI 1.041–3.993, p = 0.038). Additional adjustments were made for confounding variables such as sex, age, household income, education, smoking, alcohol consumption, walking activity, abdominal obesity, hypertension, low HDL cholesterol, elevated triglycerides, hyperglycemia, fT4, and TPOAb. Subclinical hypothyroidism remained a significant risk factor for CKD, even after adjustments (OR 2.161, 95% CI 1.032–4.527, p = 0.041).

**Table 3:** Odds ratios (ORs) and 95% confidence intervals (CIs) for chronic kidney disease based on thyroid function status

	Subclinical hyperthyroidism (n = 77, 2.5%)		Euthyroidism (n = 3,069, 94.0%)	Subclinical hypothyroidism (n = 111, 3.5%)
Chronic kidney disease				
Model 1		0.211 (0.044–1.014)	1.000	2.039 (1.041–3.993)*
Model 2		0.216 (0.044–1.047)	1.000	2.178 (1.030–4.604)*
Model 3		0.218 (0.046–1.036)	1.000	2.161 (1.032–4.527)*

Model 1, unadjusted; Model 2, with adjustment for age and sex; Model 3 as model 2, with additional adjustment for household income, education, smoking, alcohol consumption, walking activity, abdominal obesity, hypertension, low high-density lipoprotein cholesterol, elevated triglycerides, hyperglycemia, free thyroxine and thyroid-peroxidase antibody

*p < 0.05

## Discussion

In the present study, we found that subclinical hypothyroidism independently predicted CKD in the general population and was associated with increased probability of CKD after adjusting for sex, age, household income, education, smoking, alcohol consumption, walking activity, abdominal obesity, hypertension, low HDL cholesterol, elevated triglycerides, hyperglycemia, fT4, and TPOAb.

Several studies have reported an association between subclinical thyroid dysfunction, particularly subclinical hypothyroidism, and CKD in the general population [8–15]. Some studies have identified subclinical thyroid hypothyroidism as a risk factor for CKD [12–15]. In a Norwegian population-based study of adults aged ≥ 40 years, CKD was more common in people with subclinical hypothyroidism, and increase in serum TSH levels within the reference range was negatively associated with eGFR [12]. A Taipei City-based cohort study of elderly adults showed that subclinical hypothyroidism was associated with a greater risk of incident CKD [13]. In a cross-sectional analysis of the Brazilian Longitudinal Study of Adult Health of adults aged 35–74 years, subclinical hypothyroidism was associated with an increased risk of CKD [14]. In a large cohort of Taiwanese individuals aged ≥ 20 years, subclinical hypothyroidism was independently associated with reduced eGFR in a dose-dependent manner [15]. However, other studies found no association between subclinical thyroid dysfunction and CKD [8–11]. In an Australian study of community-dwelling older adults aged ≥ 60 years, increasing serum TSH levels were associated with a greater likelihood of prevalent CKD, but no significant association was observed between subclinical hypothyroidism and prevalent CKD [8]. A cross-sectional study including male participants in China showed that TSH was negatively associated with eGFR, but the prevalence of CKD was only significantly higher in participants with TSH level exceeding 7.0 mIU/L [9]. A recent study in a US community-based population of middle-aged adults demonstrated no statistically significant association between subclinical hypothyroidism and the prevalence of CKD [11]. These inconsistent results among studies may be due to differences in the definition of subclinical thyroid dysfunction and CKD as well as differences in the characteristics of the population analyzed, such as age, sex, and region, and adjustments for covariates. Although the prevalence of subclinical hypothyroidism is highly dependent on the applied TSH cut-off, subclinical hypothyroidism was defined differently in each study as TSH levels exceeding 3.50 [12], 4.00 [8–10, 14], or 5.00 mIU/L [11, 13, 15]. Albuminuria is a strong predictor of CKD [24]; however, in most studies [8–12], CKD was defined by eGFR alone using the Modification of Diet in Renal Disease formula or CKD-EPI formula. Two other studies [13, 15] defined CKD as eGFR and/or semi-quantitative measurement of proteinuria using dipstick grading. The main advantage of our study over previous studies is that we used TSH reference ranges for Koreans and quantitative measurements of proteinuria, although not a 24-hr urine collection, and adjusted for confounding factors, including socioeconomic, medical and laboratory factors. Consequently, we found an association between subclinical hypothyroidism and CKD similar to previous studies [12–15]. On the other hand, when we divided CKD into albuminuria and eGFR in this study, we found a statistically significant difference in albuminuria among the three groups according to thyroid function status, but no statistically significant difference in eGFR. And only 9.6% of the participants with albuminuria had overt proteinuria. Since changes in albuminuria is a sensitive measure that can detect kidney damage in its early stage, even before a significant decline in eGFR < 60 mL/min/1.73m^2^ [25], it is possible that only albuminuria showed a significant difference according to subclinical thyroid dysfunction.

Both CKD and subclinical hypothyroidism are known to be associated with CVD risk and increased mortality [1, 26]. Therefore, we adjusted for several metabolic risk factors in this study, and subclinical hypothyroidism was found to be independently associated with CKD. The mechanisms underlying the association between subclinical hypothyroidism and CKD remain to be elucidated. Subclinical hypothyroidism may worsen kidney function through direct and indirect effects such as reductions in cardiac output and renal blood flow, increases in systemic vascular resistance, intrarenal vasoconstriction and alterations in glomerular structure [27]. Thyroid hormone replacement therapy in CKD patients with subclinical hypothyroidism attenuates the rate of eGFR decline [28]. In addition, endothelial dysfunction has been consistently observed in CKD patients of all age groups [29] and patients with subclinical hypothyroidism have been reported to exhibit endothelial dysfunction [30]. Therefore, it seems reasonable to suggest an association between subclinical hypothyroidism and CKD. Further investigation is required in this field.

On the other hand, eGFR and renal blood flow are known to increase in patients with hyperthyroidism [6]. However, similar to previous studies [8–12], no significant association between subclinical hyperthyroidism and CKD was observed in this study.

Despite the strength of a nationally representative large cohort from the KNHANES and the control of extensive data on several potential confounding factors, including socioeconomic status indicators and medical co-morbidities, our study has some limitations. Due to the cross-sectional design, a causal relationship between subclinical thyroid dysfunction and CKD could not be inferred. Although we comprehensively adjusted for possible confounding factors, a longitudinal study is required to address this issue. Subclinical thyroid dysfunction and alteration in kidney function may be temporary, and repeated measurements of thyroid and kidney functions could provide reliable results. A single measurement may have resulted in the inclusion of transient subclinical thyroid dysfunction or transient decline in kidney function. Due to the lack of a detailed medical history in the KNHANES, secondary causes of CKD, such as polycystic kidney disease or glomerular disease, could not be considered. In addition, several types of medication, such as non-steroidal anti-inflammatory drugs, may interfere with kidney function; however, data regarding medication history are limited.

## Conclusion

Our findings demonstrated that subclinical hypothyroidism is associated with CKD, independent of known metabolic risk factors, in the general population. From a CVD prevention perspective, some patients with subclinical hypothyroidism and CKD may benefit from replacement therapy. Further studies on subclinical hypothyroidism and CKD are warranted.

## Electronic supplementary material

Below is the link to the electronic supplementary material.

Supplementary Material 1 Supporting information files_data set1

Supplementary Material 2 Supporting information files_data set2

## Data Availability

Data described in the manuscript, code book, and analytic code will be made publicly and freely available without restriction at https://knhanes.kdca.go.kr/knhanes/main.do.

## Abbreviations

CKD: chronic kidney disease
CVD: cardiovascular disease
KNHANES: Korea National Health and Nutrition Examination Survey
KCDC: Korean Centers for Disease Control and Prevention
TSH: thyroid-stimulating hormone
fT4: free thyroxine
TPOAb: thyroid-peroxidase antibody
BMI: body mass index
eGFR: estimated glomerular filtration rate
CKD-EPI: Chronic Kidney Disease Epidemiology Collaboration
HDL: high-density lipoprotein
HbA1c: glycated hemoglobin
ACR: albumin-creatinine ratio
OR: odds ratio
CI: confidence interval.

## References

[1] Global regional, national burden of chronic kidney disease (2020). 1990–2017: a systematic analysis for the global burden of Disease Study 2017. Lancet (London England).

[2] Coresh J, Astor BC, Greene T, Eknoyan G, Levey AS (2003). Prevalence of chronic kidney disease and decreased kidney function in the adult US population: Third National Health and Nutrition Examination Survey. Am J kidney diseases: official J Natl Kidney Foundation.

[3] Zhang L, Wang F, Wang L, Wang W, Liu B, Liu J, Chen M, He Q, Liao Y, Yu X (2012). Prevalence of chronic kidney disease in China: a cross-sectional survey. Lancet (London England).

[4] Stanifer JW, Jing B, Tolan S, Helmke N, Mukerjee R, Naicker S, Patel U (2014). The epidemiology of chronic kidney disease in sub-saharan Africa: a systematic review and meta-analysis. The Lancet Global health.

[5] Brück K, Stel VS, Gambaro G, Hallan S, Völzke H, Ärnlöv J, Kastarinen M, Guessous I, Vinhas J, Stengel B (2016). CKD prevalence varies across the European General Population. J Am Soc Nephrology: JASN.

[6] Iglesias P, Díez JJ (2009). Thyroid dysfunction and kidney disease. Eur J Endocrinol.

[7] Jabbar A, Pingitore A, Pearce SH, Zaman A, Iervasi G, Razvi S (2017). Thyroid hormones and cardiovascular disease. Nat reviews Cardiol.

[8] Gopinath B, Harris DC, Wall JR, Kifley A, Mitchell P (2013). Relationship between thyroid dysfunction and chronic kidney disease in community-dwelling older adults. Maturitas.

[9] Ye Y, Gai X, Xie H, Jiao L, Zhang S (2013). Impact of thyroid function on serum cystatin C and estimated glomerular filtration rate: a cross-sectional study. Endocr practice: official J Am Coll Endocrinol Am Association Clin Endocrinologists.

[10] Chaker L, Sedaghat S, Hoorn EJ, Elzen WP, Gussekloo J, Hofman A, Ikram MA, Franco OH, Dehghan A, Peeters RP (2016). The association of thyroid function and the risk of kidney function decline: a population-based cohort study. Eur J Endocrinol.

[11] Schultheiss UT, Daya N, Grams ME, Seufert J, Steffes M, Coresh J, Selvin E, Köttgen A. Thyroid function, reduced kidney function and incident chronic kidney disease in a community-based population: the Atherosclerosis Risk in Communities study. *Nephrology, dialysis, transplantation: official publication of the European Dialysis and Transplant Association - European Renal Association* 2017, 32(11):1874–1881. 10.1093/ndt/gfw301 PMC5837276 27540046

[12] Asvold BO, Bjøro T, Vatten LJ (2011). Association of thyroid function with estimated glomerular filtration rate in a population-based study: the HUNT study. Eur J Endocrinol.

[13] Chuang MH, Liao KM, Hung YM, Wang PY, Chou YC, Chou P (2016). Abnormal thyroid-stimulating hormone and chronic kidney Disease in Elderly adults in Taipei City. J Am Geriatr Soc.

[14] de Peixoto ÉJF, Bittencourt MS, Goulart AC, Santos IS, de Oliveira Titan SM, Ladeira RM, Barreto SM, Lotufo PA, Benseñor IJM (2017). Thyrotropin levels are associated with chronic kidney disease among healthy subjects in cross-sectional analysis of the brazilian longitudinal study of Adult Health (ELSA-Brasil). Clin Exp Nephrol.

[15] Chang YC, Chang CH, Yeh YC, Chuang LM, Tu YK (2018). Subclinical and overt hypothyroidism is associated with reduced glomerular filtration rate and proteinuria: a large cross-sectional population study. Sci Rep.

[16] Kim WG, Kim WB, Woo G, Kim H, Cho Y, Kim TY, Kim SW, Shin MH, Park JW, Park HL (2017). Thyroid stimulating hormone reference range and prevalence of thyroid dysfunction in the Korean Population: Korea National Health and Nutrition Examination Survey 2013 to 2015. Endocrinol metabolism (Seoul Korea).

[17] Park S, Kim WG, Jeon MJ, Kim M, Oh HS, Han M, Kim TY, Shong YK, Kim WB (2018). Serum thyroid-stimulating hormone levels and smoking status: data from the Korean National Health and Nutrition Examination Survey VI. Clin Endocrinol.

[18] Sanyal AJ, Brunt EM, Kleiner DE, Kowdley KV, Chalasani N, Lavine JE, Ratziu V, McCullough A (2011). Endpoints and clinical trial design for nonalcoholic steatohepatitis. Hepatology (Baltimore MD).

[19] Lee YH, Kim SU, Song K, Park JY, Kim DY, Ahn SH, Lee BW, Kang ES, Cha BS, Han KH (2016). Sarcopenia is associated with significant liver fibrosis independently of obesity and insulin resistance in nonalcoholic fatty liver disease: nationwide surveys (KNHANES 2008–2011). Hepatology (Baltimore MD).

[20] Kim HJ, Park SJ, Park HK, Byun DW, Suh K, Yoo MH (2021). Thyroid autoimmunity and metabolic syndrome: a nationwide population-based study. Eur J Endocrinol.

[21] Kim HJ, Park SJ, Park HK, Byun DW, Suh K, Yoo MH (2022). Association of free thyroxine with obstructive lung pattern in euthyroid middle-aged subjects: a population-based study. PLoS ONE.

[22] Appropriate body-mass (2004). Index for asian populations and its implications for policy and intervention strategies. Lancet (London England).

[23] Levey AS, Stevens LA, Schmid CH, Zhang YL, Castro AF, Feldman HI, Kusek JW, Eggers P, Van Lente F, Greene T (2009). A new equation to estimate glomerular filtration rate. Ann Intern Med.

[24] Stevens PE, Levin A (2013). Evaluation and management of chronic kidney disease: synopsis of the kidney disease: improving global outcomes 2012 clinical practice guideline. Ann Intern Med.

[25] Gansevoort RT, Correa-Rotter R, Hemmelgarn BR, Jafar TH, Heerspink HJ, Mann JF, Matsushita K, Wen CP (2013). Chronic kidney disease and cardiovascular risk: epidemiology, mechanisms, and prevention. Lancet (London England).

[26] Moon S, Kim MJ, Yu JM, Yoo HJ, Park YJ (2018). Subclinical hypothyroidism and the risk of Cardiovascular Disease and all-cause mortality: a Meta-analysis of prospective cohort studies. Thyroid: official journal of the American Thyroid Association.

[27] Mariani LH, Berns JS (2012). The renal manifestations of thyroid disease. J Am Soc Nephrology: JASN.

[28] Shin DH, Lee MJ, Lee HS, Oh HJ, Ko KI, Kim CH, Doh FM, Koo HM, Kim HR, Han JH (2013). Thyroid hormone replacement therapy attenuates the decline of renal function in chronic kidney disease patients with subclinical hypothyroidism. Thyroid: official journal of the American Thyroid Association.

[29] Fliser D, Wiecek A, Suleymanlar G, Ortiz A, Massy Z, Lindholm B, Martinez-Castelao A, Agarwal R, Jager KJ, Dekker FW (2011). The dysfunctional endothelium in CKD and in cardiovascular disease: mapping the origin(s) of cardiovascular problems in CKD and of kidney disease in cardiovascular conditions for a research agenda. Kidney Int supplements.

[30] Cikim AS, Oflaz H, Ozbey N, Cikim K, Umman S, Meric M, Sencer E, Molvalilar S (2004). Evaluation of endothelial function in subclinical hypothyroidism and subclinical hyperthyroidism. Thyroid: official journal of the American Thyroid Association.

